# Essential Medicines for Dental Caries: Cost-Effectiveness of ART and SDF: Julia Gierth

**DOI:** 10.1093/eurpub/ckac131.010

**Published:** 2022-10-25

**Authors:** J Gierth, J Coughlan, N Gkekas, T Hofbauer, S Listl, M Pattamatta, A Pold

**Affiliations:** 1 Dentistry, University of Heidelberg, Heidelberg, Germany; 2 Global Health, Heidelberg Graduate School of Global Health, Heidelberg, Germany; 3 Quality and Safety of Oral Health Care, Radboud University, Nijmengen, Netherlands; 4 Health Sciences, University of York, York, UK; 5 Health Science, Queen Mary University of London, London, UK

## Abstract

**Background:**

Untreated tooth decay (caries) is the most common global health condition and one of the largest preventable disease burdens for society. It concerns both children and adults, particularly in low resource settings whereout of pocket expenditures for oral care often cause catastrophic health expenditures. The 2021 WHO Oral Health Resolution emphasized the relevance of developing so-called “Best Buys” for oral health. The purpose of this study was to identify the cost-effectiveness of Atraumatic Restorative Treatment (ART) and Silver Diamine Fluoride (SDF) as potential treatments to reduce the caries burden worldwide.

**Methods:**

Leaning on WHO CHOICE methodology, evidence scoping and an expert consensus were facilitated to extract model input parameters which were then fed into cost-effectiveness-analyses (CEA) for ART and SDF. The cost-effectiveness of the interventions was expressed as Cost per DALY averted.

**Results:**

The evidence scoping revealed relevant, information on the effectiveness and costs of ART and SDF. The CEA identified both ART and SDF to be potentially cost-efficient treatment strategies in settings with limited resources. SDF was found to provide a cost-efficient treatment alternative in settings where the comparably larger (human) resource requirements for ART cannot be met.

**Conclusions:**

The findings suggest that ART and SDF represent potentially cost-efficient strategies to reduce the caries burden in settings with limited resources. While ART has previously been proposed as part of WHO’s essential package of oral care, SDF could provide a comparably inexpensive treatment alternative.

**Key messages:**

• Untreated tooth decay (caries) is the most common global health condition and one of the largest preventable disease burdens for society.

• The CEA identified both ART and SDF to be potentially cost-efficient treatment strategies in settings with limited resources.

